# Carinal surgery: experience of a single center and review of the current literature

**DOI:** 10.1186/1749-8090-5-51

**Published:** 2010-06-19

**Authors:** Haralabos Parissis, Vincent Young

**Affiliations:** 1 Cardiothoracic Department, Royal Victoria Hospital, Belfast, Northern Ireland; 2 Cardiothoracic Department, St James Hospital, Dublin 8, Dublin, Republic of Ireland

## Abstract

**Background:**

To report our experience for the treatment of lung tumors of the right main bronchus (RMB) invading the carina.

**Methods:**

From February 2000 till January 2007 we have identified 8 cases (1.09%) requiring carinal surgery.

Plan of action: Close cooperation with anaesthetics, long flexible ET tube, Right posterolateral thoracotomy, no irrevocable steps until resection guaranteed, mobilization of trachea and main bronchus, division of the trachea & Left main bronchus. Intubate across surgical field. Tailoring for airway size discrepancies, appropriately. Construction of the tracheobronchial anastomosis around the ventilatory tube. Skillfull reintubation, over a long boogie.

**Results:**

Mortality: 12.5% due to ARDS (one patient)

Morbidity: anastomotic stenosis requiring stent (one patient). Follow-up 52 ± 11 months.

Recurrences: 2 patients (both with pathological N2 disease on histology).

**Conclusions:**

Success of carinal surgery depends on careful patient selection, team approach and attention to detail. Patients with N2 disease carry the worst prognosis.

## Background

Until recently the TNM classification staged tumours invading the carina as T4, IIIB. By implication these tumours are inoperable due to local criteria. However a subgroup of those patients can be treated by carinal resection and reconstruction with potential cure. Therefore, there is an argument that this subgroup should the staged as IIIA[1]; especially since this small group of patients consists of a potentially surgical group with favourable outcome and a five years survival up to 40%-45% [2].

Indications for carinal resection are reported [3] as: bronchogenic carcinoma (43.2%), other airway neoplasms (44.7%) and benign or inflammatory strictures (11.9%).

In this report we are presenting our experience with right side carinal pneumonectomy and carina plasty. The indications, surgical steps and early outcome are reported.

## Methods

Our series of carinal surgery consists of a small number of patients performed by a single surgeon (the senior author of the paper, VY).

From February 2000 up till January 2007 we have identified 8 cases (1.09% of all pulmonary resections) of carinal surgery in our institution: 5 cases of right side carinal sleeve pneumonectomy (CSP) and 3 cases of carinal plasty (resection of the carina with preservation of both left and right lung).

CSP was considered when there was suitable anatomy, clinical N0, N1 disease, stump recurrence following lobectomy or positive margins after right side pneumonectomy and carcinoid/sarcoma of the carina. Written informed consent was obtained from the patients for this publication.

### Left carinal pneumonectomy was not encountered in this series

**Figure 1:**
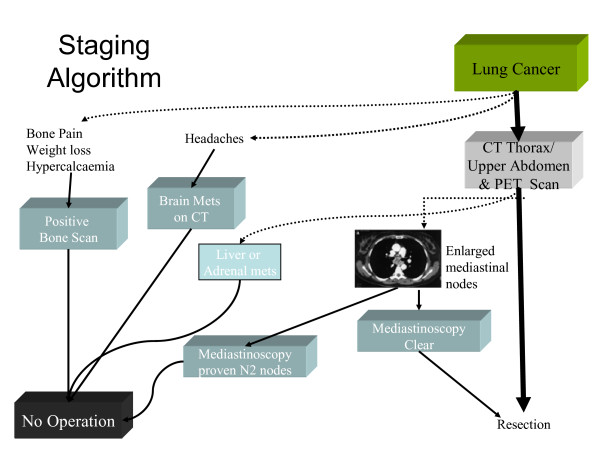
Staging algorithm for patients prior to carinal resection.

The rate of false negative pre-operative CT evaluation of the mediastinum could be up to 35%; moreover the sensitivity and specificity of CT staging of the mediastinal nodal involvement is 78% [4]. For those reasons, all the patients in these series had undergone PET scan and mediastinoscopy (Figure 1).

Preoperative N2 disease was a contraindication for carina surgery. Therefore neoadjuvant downstaging was not considered in those series.

Brain CT in our institution is carried out only when there are clinical signs or symptoms of brain metastasis. Likewise bone scan is deemed necessary only when bone pain, hypercalcaemia or high alk phosphatase is present.

**Figure 2:**
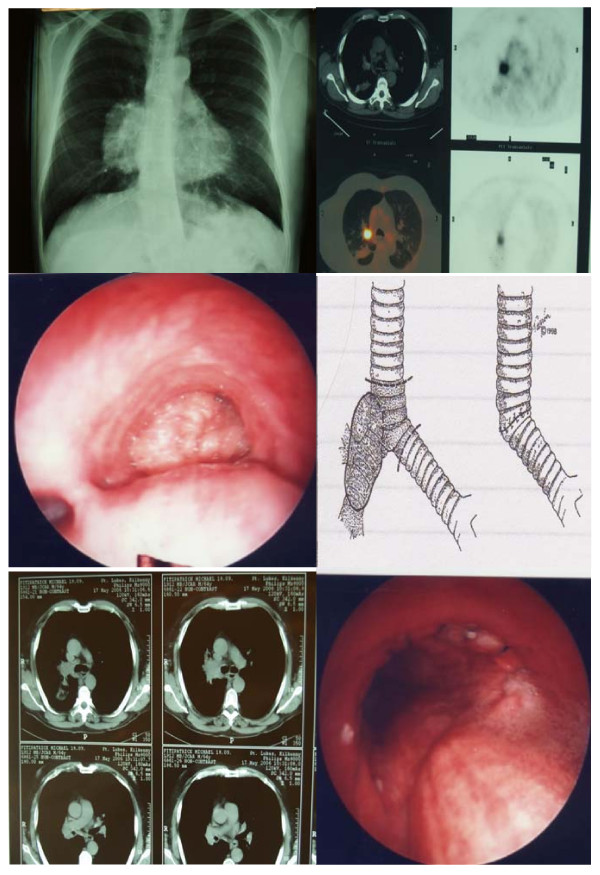
**A case of a central Squamous cell carcinoma of the Right upper lobe invading the carina**. CXR, CT Scan of the chest, PET scan, schematic representation of the tumor and Bronchoscopy before and after carinal resection showing tracheo-bronchial anastomosis.

Assessment by rigid bronchoscopy and biopsy of the lower trachea/carina/right main bronchus is also carried out to further delineate the extent of the tumor involvement (Figure 2).

A favourable outcome following CSP is feasible if optimal anaesthetic and surgical strategies are to be taken into consideration.

### Anaesthetic strategies

Multidisciplinary team approach and close collaboration with the anaesthetist, is required.

Long flexible Endo Tracheal (ET) tube, is used. No irrevocable steps are taken until the resection is guaranteed. Intraoperative barotrauma has to be eliminated by avoiding long periods of collapsed lung. Finally, after the posterior part of the tracheobronchial anastomosis has been constructed, skillfull reintubation over a long boogie is required.

### Surgical strategies

We advocate right posterolateral thoracotomy through the 5^th ^intercostal space.

Mediastinal lymph node resection (stations R2, 4, 7, 8, 9) is carried out routinely in our department. The lower trachea and left main bronchus (LMB) are mobilized. The exposure is usually better facilitated, following dissection and removal of station 7 lymph nodes. Bronchial vessels in the subcarinal region are dealt with diathermia.

We use traction sutures on the lower trachea and a tape around the LMB, prior to division. Following division of the LMB, we ensure across surgical field intubation with a small size ET tube to maintain ventilation of the left lung. Frozen sections are only required if R0 resection margins are "macroscopically questionable" (none of our reported cases). Up to two tracheal rings can be removed without a danger of putting the anastomosis under tension. The construction of the end to end or end to side airway anastomosis is fashioned with a continuous 3.0 prolene. The back wall is completed first, following by endotracheal intubation across the anastomosis. The front wall is then done over the long ET tube; care must be taken to avoid "incorporating the ET tube" on the suture line. Flexible Bronchoscopic inspection of the suture line is performed routinely. Traditionally in our institution, we used prolene suture for all bronchial surgery (closure of a stump, sleeve lobectomy or bronchoplasty procedures). Discrepancies in size are less of a problem than in simple sleeve lobectomy; but where encountered were dealt with by careful adjustment of suture placement on the two airways. Telescopic anastomotic technique due to differences in size was not encountered in those series. Furthermore, we did not circumferentially wrap the anastomosis with any viable tissue (eg. Omentum or intercostal muscle).

Finally, specific release manoeuvres are not required but there is considerable mobilisation of structures due to the complete mediastinal nodal dissection performed in all patients. This is an important technical as well as oncological component of the operation.

## Results

Our experience with carinal surgery, consists of 8 patients over a 7 year period. There were relative younger patients (mean age 58 ± 3 years) compare to the age of the overall lung resection patients (67 ± 8.3 years). The patients were predominantly males with the histological diagnosis of Squamous cell Carcinoma (Table 1). One patient underwent a completion sleeve pneumonectomy: he was a 57 year old male who in a routine follow up 5 years following Right middle & lower Lobectomy for NSCLC he was found to have collapsed of the residual lung. Bronchoscopy showed NSCLC involving the origin of the right main bronchus. He underwent a completion pneumonectomy with carinal resection. Finally, three patients underwent carinal-plasty for tracheal sarcoma or carcinoid tumours.

**Table 1:** Patients characteristics

Pt	1	2	3	4	5	6	7	8
**Age**	58	62	55	55	62	66	64	25

**Sex**	M	M	M	M	F	M	F	M

**Procedure**	RSP	RSP	RSP	RCSP	CP	RSP	CP	CP

**LOS(days)**	19	18	28	16	12	16	9	11

**Complications**	Chest infestion	Anastomotic stenosis	ARDS	-	-	arrhytmias	-	-

**Histology**	SCC	SCC	SCC	SCC	TS	SCC	Carcinoid	Carcinoid

**TNM**	T4N0M0	T4N2M0	T4N1 M0	T4N1 M0	N0	T4N2 M0	N0	N0

**Recurrence**	No	Yes	No	No	No	Yes	No	No

**Status**	A	A	D	A	A	A	A	A

**Survival (months)**	A (50)	D (12)	D (1)	A (39)	A (29)	D (3)	A (26)	A (28)

SCC: Squamous cell Carcinoma, RSP: Right Sleeve pneumonectomy, RCSP: completion sleeve pneumonectomy for T1N0 tumor removed with R middle & lower lobectomy 5 years ago.

CP: carinal plasty with reinplantation of the right main bronchus to the trachea for Tracheal Sarcoma (TS) or carcinoid tumor

A:Alive

D:Dead

One patient died due to respiratory failure and ARDS two weeks following surgery. One patient developed anastomotic stenosis manifested five weeks postoperatively with stridor. He required dilatation and stenting across the anastomosis. Two patients (both with pathological N2 disease on histology) developed recurrences: patient number 2 developed local recurrences 1 year later and patient number 6 re-presented with brain metastasis 3 months after surgery.

Two patients with SCC were the long term survivors following RSP at 50 and 39 months. Finally, the only patient with the Tracheal sarcoma was alive at 29 months and the 2 patients with the carcinoid tumors were alive at 26 and 28 months of follow up.

## Discussion

Pathological processes that involve the carina pose a challenge to the thoracic surgeons.

Patients must be able to withstand the procedure, and they must be told that the operative mortality is 2 to 4 times higher than what is expected after standard pneumonectomy [5]. Nevertheless, techniques have been developed to allow primary resection and reconstruction with relative moderate risk and a five year survival that does not really reflect stage IIIB disease.

Our experience refers to limited number of cases, however useful thoughtful suggestions could be derived out of it; those technically demanding operations are requiring team approach and a sound decision making process.

Maintaining optimal oxygenation through out the procedure is desirable. There are various ventilation options during construction of the airway anastomosis. Cross field ventilation is the most common used technique but requires close anaesthetic collaboration. Apnoeic oxygenation operates on the principle that with preoxygenation and hyperventilation 10-12 min of total apnea can be safely tolerated. However, due to hypoxic complications a modified technique is usually advocated whereby hyperoxygenation is followed by cross surgical field ventilation with a 10F catheter and delivering 15 L/min O2. Finally, high Frequency Jet Ventilation delivered through a small bored catheter is infrequently implemented.

If the SVC is involved (up to 20% of the cases) then a brain protection strategy during concomitant SVC surgery is required. We advocate monitoring of the jugular bulb pressure (JBP) and we aim to optimise cerebral perfusion pressure by increasing the MAP at least to 60 mmHg above the JBP. We also aim to keep the jugular bulb oxygen saturation greater than 50%. Neither venous shunt from the brachiocephalic vein to the right atrium nor neuro-protective agents and mannitole has been used by our group.

In order to achieve R0 resection margins, careful planning is essential. Accurate bronchoscopic biopsies along the carina are obtained; distal sides are biopsied first. Nevertheless, surgeons must always remember that it is better and safer to accept a positive resection margin than to have to deal with a bronchopleural fistula caused by anastomotic separation [5]. This is because anastomotic complications are often life-threatening [6]. The most feared complication however, is postoperative adult respiratory distress syndrome [7] encountered in up to 11% of the cases.

The postoperative mortality was 12.5% in our series due to ARDS. Mitchell et al [8] reported a 20% mortality for Carinal Pneumonectomy and 11% for carinal plasty. The overall mortality is high and this could be explained by looking at the patients characteristics (Table 2): up to 20% of the patients in those series had previous lung surgery and also up to 35% had previous chemoradiation for downstaging [9-11].

**Table 2:** Characteristics and outcome following surgery for carinal pathology

**Patients Characteristics **[8,9]	**Histology **[8,10]	Complications 36-45%	Concomitant procedures	Mortality Mainly due to MI, ARDS, PE	5 year survival
Predominantly males(2/3)	SCCa 70%-77%	Arrhythmia 18.3%	SVC Surgery 22%	4% [2]	15% [12]

Previous lung surgery: 14-21.6%	AdenoCa 18%-20%	Anastomotic leak 16.7%	Excision of diaphragm 3%	15% [8]	33.4% [13]

Previous chemoradiation: 15-36%	Large Cell 2- 7%	ARDS 10%	Chest wall 2%	4% [9]	42% [8]

Carinal pneumonectomy: 58-68% Carinal Plasty: 30% Stump revision: 4%	Mixed 1-3%	Pneumonia 6.7%	LA 1%	7.6% [11]	44% [11]

References: [2,8-13]

SCCa: Squamous Cell Carcinoma

SVC: Superior Vena Cava

LA: Left Atrium

MI: Myocardial Infarction

ARDS: Adult respiratory distress Syndrome

PE: Pulmonary Embolism

Predictors of operative mortality included postoperative mechanical ventilation, length of resected airway and development of anastomotic complications [3].

The 5 year survival (Table 2) was low in the early series [12] however late reports are quoting survival up to 44%[11,13];Moreover Mitchell et al [3] has calculated the 5 year survival according to the pathology of the disease to be : 38% for Carinal Pneumonectomy and up to 51% for carinal plasty. In our report out of 5 patients following CSP, there was one operative death and 2 recurrences within the first year. Two patients were intermediate survivors (e.g. alive 3-4 years later). Moreover, the 3 patients that underwent carinal plasty were still alive 2 and half years later.

Tumour recurrences are only mentioned in few reports [10,11]; the recurrence rate in our series was 25% but it has been quoted to be as high as 75% [14]. Furthermore we observe a high local and distal recurrence rate in patients with pathological N2 disease; this probably outlines aggressive disease and therefore a very close follow up and post operative chemoradiation may be justified.

Nodal status Influences of the outcome according to various reports [8,10,11,15,16]: patients with pathologically N2 disease have a 12% 5 year survival versus 53% for N0.

The role of neoadjuvant Chemo-Radiation therapy is debatable: According to some reports [9] it downstages 40% of N2 nodes therefore increases the pool of patients that they would benefit from surgical resection; However, according to other reports [17] this type of therapy should be used with caution because of the deleterious effects on anastomotic healing.

Radical lymphadenectomy is advocated in our centre routinely. This was supported by other groups. The high incidence of micrometastatic nodes in positron emission tomography-negative patients according to Macchiarini et al [9] justifies routine mediastinoscopy and radical lymphadenectomy.

Finally is Sleeve pneumonectomy a justifiable procedure? The answer is negative, if the mortality rate is similar to the long-term survival and positive, if one can achieve an operative mortality under 10% and a five year survival over 20%.

## Conclusions

The various techniques of carinal surgery could be applied in selective cases with optimal outcome [18,19].

However, success depends on careful patient selection, attention to detail and accurate preoperative staging [20].

The results reflect the technical complexity of the operation and the natural history of lung cancer but, five year survival in excess of 40% for malignant disease may be anticipated in the absence of involved mediastinal lymph nodes.

## Competing interests

The authors declare that they have no competing interests.

## Authors' contributions

HP participated in the sequence alignment and drafted the manuscript and VY participated in its design and coordination. The authors read and approved the manuscript.
